# Determination of Vehicle Trajectory through Optimization of Vehicle Bounding Boxes using a Convolutional Neural Network

**DOI:** 10.3390/s19194263

**Published:** 2019-09-30

**Authors:** Seonkyeong Seong, Jeongheon Song, Donghyeon Yoon, Jiyoung Kim, Jaewan Choi

**Affiliations:** CAL Lab., HyperSensing Inc., Yuseong-gu, gwahak-ro, Daejeon 169-84, Korea; skseong@hypersensing.net (S.S.); newssong@hypersensing.net (J.S.); dhyoon@hypersensing.net (D.Y.);

**Keywords:** vehicle trajectory, convolutional neural network, YOLOv2, Kalman filter

## Abstract

In this manuscript, a new method for the determination of vehicle trajectories using an optimal bounding box for the vehicle is developed. The vehicle trajectory is extracted using images acquired from a camera installed at an intersection based on a convolutional neural network (CNN). First, real-time vehicle object detection is performed using the YOLOv2 model, which is one of the most representative object detection algorithms based on CNN. To overcome the inaccuracy of the vehicle location extracted by YOLOv2, the trajectory was calibrated using a vehicle tracking algorithm such as a Kalman filter and intersection-over-union (IOU) tracker. In particular, we attempted to correct the vehicle trajectory by extracting the center position based on the geometric characteristics of a moving vehicle according to the bounding box. The quantitative and qualitative evaluations indicate that the proposed algorithm can detect the trajectories of moving vehicles better than the conventional algorithm. Although the center points of the bounding boxes obtained using the existing conventional algorithm are often outside of the vehicle due to the geometric displacement of the camera, the proposed technique can minimize positional errors and extract the optimal bounding box to determine the vehicle location.

## 1. Introduction

The traffic conditions of downtown or urban spaces have become a very important issue for traffic management, and intelligent transportation systems (ITSs) have been rapidly developed [1]. As image processing techniques and various terrestrial sensors have been developed, the demand for a system capable of analyzing traffic conditions has increased, and ITSs have been recognized as a key technology for smart cities. In particular, the traffic conditions can help analyze the routes of vehicles in real time; thus, it is recognized as essential information for operation of the autonomous vehicle. However, there is demand for a system to control traffic signals in real time by analyzing the amount of road traffic, such as at an intersection, but this type of system has not been firmly established due to the lack of technology for capturing traffic information. Therefore, an attempt was made to analyze the traffic conditions by collecting global navigation satellite system (GNSS) data for each vehicle; however, these data were impossible to appropriately apply to traffic signals because the analysis took too much time. Additionally, inductive loop detectors and image-based detectors are mainly used as the method for collecting traffic data based on traffic information on the road [2]. However, to apply these data to real-time traffic analysis, inductive loop detectors require considerable labor and cost for the pavement of roads due to complex installation, maintenance, and asphalt deterioration. In addition, these instruments can be damaged by wear and tear [2]. In the case of an image-based detector, there is a problem in that a corresponding number of detectors must be installed and managed to obtain traffic data from multiple lanes and directions. To solve these problems, various algorithms for extracting the vehicle position from the spatial information acquired through the sensor should be developed. Therefore, in the fields related to image processing, traffic, and autonomous vehicles, studies on traffic condition analysis and vehicle detection using images and sensors have been analyzed.

Various sensors, including magneto-impedance, magnetoresistance, and fluxgate sensors, have been developed for counting and classifying vehicles [3,4,5,6,7]. Dong et al. [8] proposed a vehicle-detection and identification algorithm based on a short-term variance sequence transformed from a magnetic signal. In addition, a vehicle-identification algorithm for four types of vehicles using 42-D features from a raw signal using a magnetic sensor is applied. Marszalek et al. [9] developed a measurement system based on an inductive loop axle detector that captures two components of a motor vehicle’s magnetic profiles

In addition, various studies based on image processing techniques have been proposed. The traffic accident recording and reporting system (ARRS) was used to analyze the occurrence of traffic accidents at intersections using a digital video recorder [10]. This system detected moving vehicles by image differencing and then extracted the speeds and locations of moving vehicles. Wang et al. [11] developed a video-image vehicle-detection system using multi-information fusion based on background modeling, color information, and texture information. Kato et al. [12] used hidden Markov models to segment vehicles, shadows, and backgrounds in an image of a motorway sequence. In addition, Cucchiara et al. [13] proposed a symbolic reasoning approach by spatiotemporal analysis. Moving edge closure in daytime frames and morphological analysis of headlights in nighttime frames were integrated for the detection of moving vehicles. A classification methodology that was robust to various weather and illumination conditions was proposed, and this methodology was based on adaptive background estimation, feature extraction, and support vector machine (SVM) [14]. Luvizon et al. [2] estimated vehicle speeds through detection feature tracking and motion prediction using scale invariant feature transform (SIFT) in regions of interest.

Recently, various algorithms based on machine learning or deep learning have been developed [15,16,17,18,19,20]. In the case of general object detection or tracking, Zhang et al. [21] detected and classified traffic signs using a deep network based on five convolutional layers and a fully connected layer on traffic-sign datasets. In addition, various visual object-tracking methods based on convolutional neural networks have been proposed for object-tracking benchmark (OTB) datasets [22,23,24].

In the case of moving-vehicle detection, Koller et al. [25] proposed a machine-learning-based system that included contour extraction of vehicles and an affine motion model using Kalman filters to extract vehicle trajectories through real-time image sequences. Wang et al. [26] generated a synthetic image for photorealistic and non-photorealistic images and then applied the transfer-learning method for vehicle detection using a faster region-convolutional neural network (Faster R-CNN). YOLOv2, which is a representative end-to-end object-detection algorithm based on CNN, was applied to vehicle detection in a single image [27]. Li et al. [28] estimated the ground speed of multiple vehicles based on a traffic dataset by unmanned aerial vehicles (UAVs) through YOLOv3 for object detection and motion compensation. In addition, Wang et el. [29] used a recurrent neural network to estimate the motion of moving objects using trajectories.

As mentioned in the related works, there are various techniques for detecting vehicles on roadways. However, vehicle detection methods by inductive loop detectors require computational costs, and the devices are expensive to maintain. In addition, detection methods based on image processing are difficult to process in real time, and accuracy improvements are required. Various deep-learning-based methods have been considered efficient algorithms for vehicle detection; however, most techniques based on deep learning, including CNN, have tracked the route of a vehicle but have been unable to determine its position accurately. Image processing techniques or deep-learning-based techniques may detect multiple vehicles as a single vehicle when two or more vehicles move via a similar path or may detect the presence of a vehicle in another lane due to the shadow of the moving vehicle. Therefore, it is relatively difficult to estimate information about the accurate path of a vehicle or the absolute position of the lane where the vehicle exists. The accurate extraction of the route of a specific vehicle would be advantageous and could be used to assist with autonomous travel policies by receiving the necessary information before reaching another intersection.

Therefore, in this manuscript, we propose a CNN-based system that can estimate the exact trajectory of a moving vehicle. First, real-time vehicle object detection is performed using the YOLOv2 model. Then, the trajectories were calibrated using a Kalman filter and intersection-over-union (IOU) tracker. In particular, we attempted to extract the accurate path of the vehicle by estimating the accurate bounding box of the moving vehicle using the basic angle and width of the front of the vehicle according to the position of the vehicle. The remainder of this manuscript is organized as follows. Section 2 describes the specifications of the camera sensor and network framework for vehicle monitoring at intersections. In Section 3, a proposed algorithm is described for the estimation of the trajectory of a moving vehicle. In Section 4, the accuracy of the proposed algorithm is evaluated and analyzed, and Section 5 concludes this study.

## 2. Sensor Platforms for Tracking Vehicle Location

### 2.1. Sensor Specification

**Figure 1:**
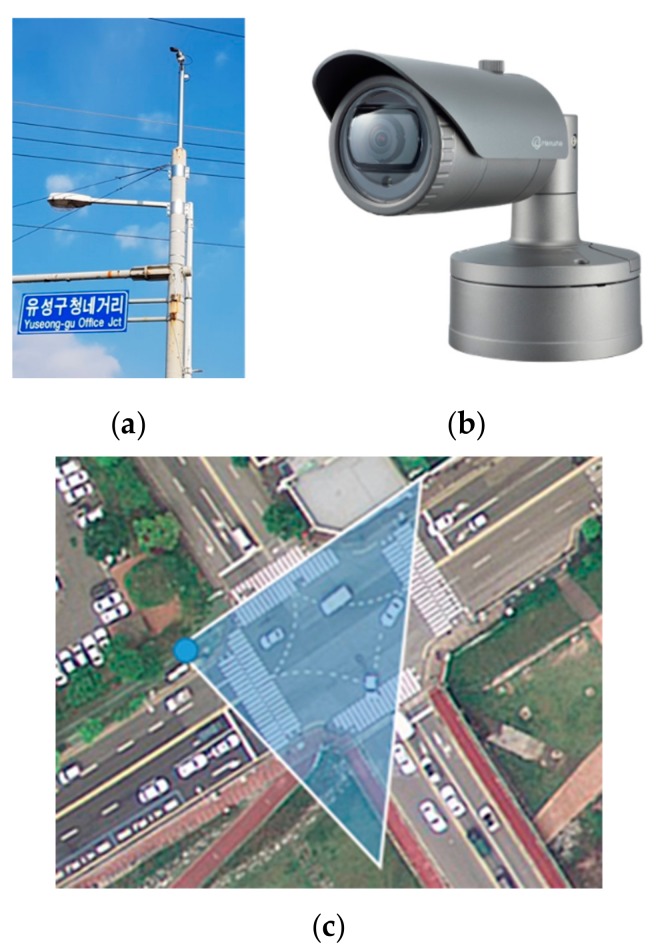
Camera location and installation in the study area: (**a**) installation of the camera; (**b**) XNO-6010R sensor; and (**c**) location and coverage of the camera at an intersection.

The system for vehicle detection consists of a closed-circuit television (CCTV) network including an optical camera sensor, an image acquisition unit, a network, and an analysis unit that receives images from the network and calculates the information related to the vehicles located in the images. A camera sensor for the acquisition of real-time images was installed as shown in Figure 1a. To collect traffic data at the intersection, the XNO-6010R sensor of Hanwha Techwin, Korea (Figure 1b) was installed on a pole at the intersection, which is located in Yuseong-gu, Daejeon, Korea. The size of the area to be captured is 33 m ×27 m, based on the center of the crosswalk (Figure 1c). To acquire data over the whole intersection area, the sensor was installed with a proper slope at a high position using a pole. The blue point in Figure 1c indicates the location where the camera was installed. The camera specifications of the XNO-6010R sensor are described in Table 1.

### 2.2. Preprocessing for the Integration of Sensor Platforms and Spatial Information

**Figure 2:**
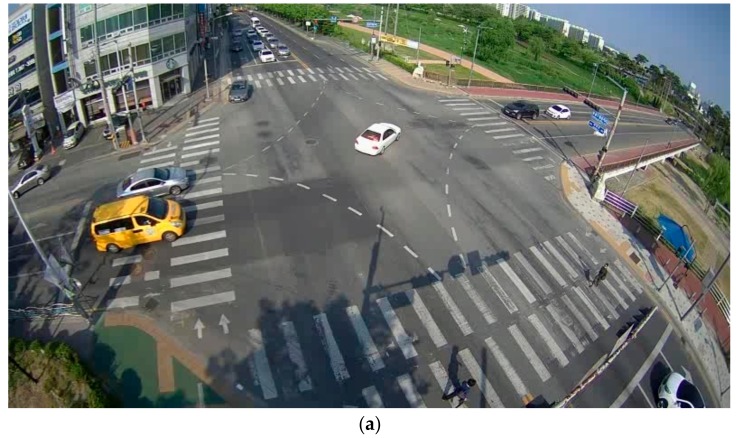

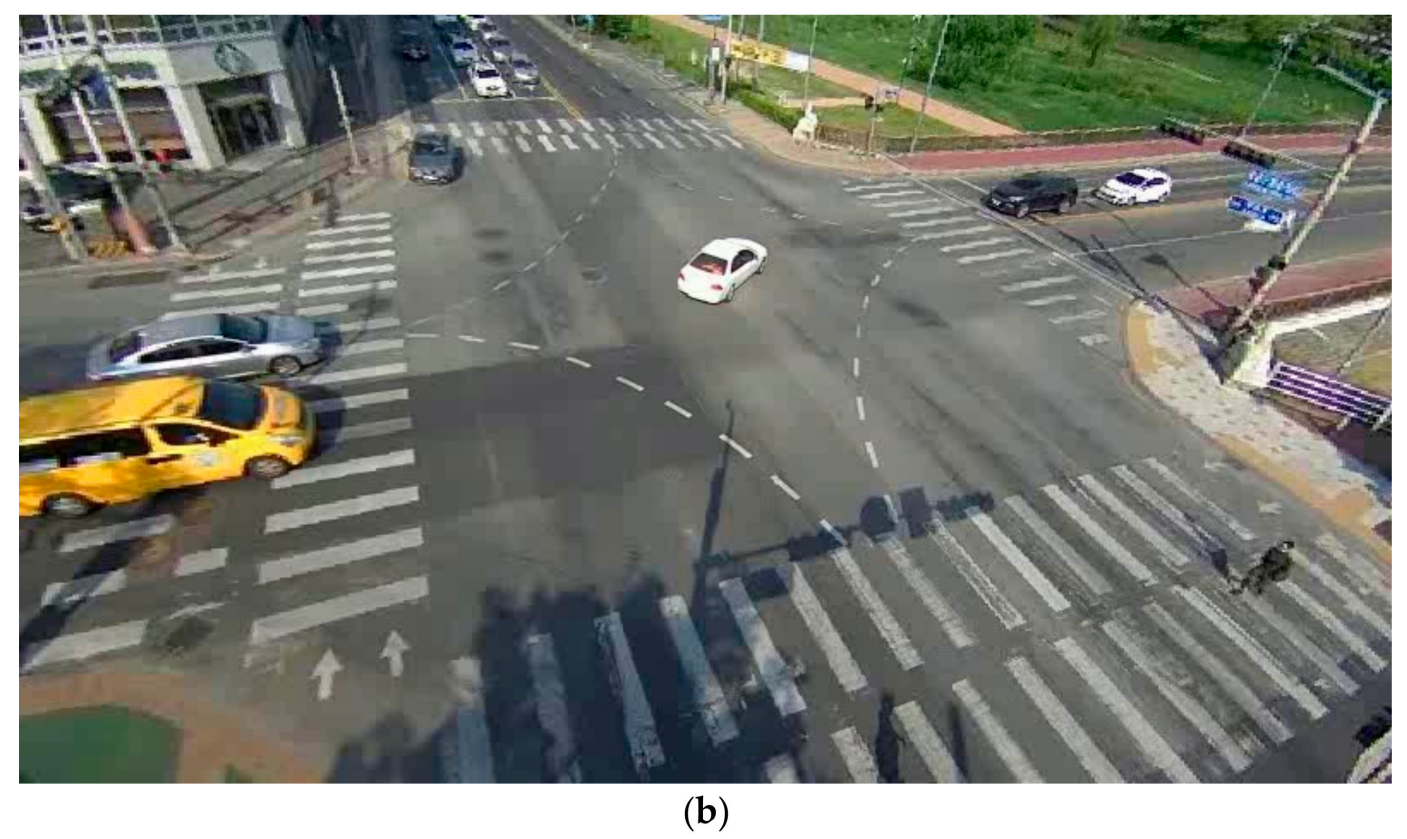
Example of lens distortion: (**a**) lens-distorted image; (**b**) image calibrated through camera calibration.

The camera sensor that was installed at the intersection has a high field of view to acquire traffic information of the entire intersection area. In addition, a non-photogrammetric sensor was also installed at the intersection. Therefore, the images collected by the camera sensor were subject to radial distortion, as shown in Figure 2a. The purpose of our study was to acquire the absolute coordinates of moving vehicles in an intersection. Therefore, in this manuscript, we corrected the images acquired through the installed camera and extracted the absolute coordinates within the intersection using a UAV image. First, the camera calibration is applied to remove the radial and tangential distortion of the image. The lens distortion of the image was estimated using Equation (1) based on the camera calibration parameter in Brown’s model [30].

(1) $$\Delta x=\bar{x}\left(K_{1}r^{2}+K_{2}r^{4}+K_{3}r^{6}\right)+\left\{P_{2}\left(r^{2}+2{\bar{x}}^{2}\right)+2P_{1}\bar{x}\bar{y}\right\}\;\Delta y=\bar{y}\left(K_{1}r^{2}+K_{2}r^{4}+K_{3}r^{6}\right)+\left\{P_{1}\left(r^{2}+2y^{2}\right)+2P_{2}\bar{x}\bar{y}\right\}$$

Here, Δx and Δy indicate the departures from collinearity due to lens distortion and $\bar{x}$ and $\bar{y}$ are the normalized image planes according to the differences between the image planes in the x and y directions and the principal point offsets $c_{x}$ and $c_{y}$. $K_{1},\;K_{2},\;\mathrm{and}\;K_{3}$ are the coefficients of radial distortion, and $P_{1}\;\mathrm{and}\;P_{2}$ are the coefficients of tangential distortion. After determination of the camera calibration parameters, the original image including lens distortion (Figure 2a) can be converted to a calibrated image, as shown in Figure 2b.

Despite the removal of the lens distortion from the image, the image captured by the camera that was installed with a proper slope at a high position using a pole had the characteristics of an oblique image. To determine the absolute coordinates of the image, reference data were generated using a UAV. The mosaiced orthophoto captured by the UAV was assumed to be an ortho-projected image. Then, the images that were acquired by the camera at the intersection were rectified by using the four tie points in the orthophoto collected by the UAV. The details are as follows.

Step (1) Select four corresponding points in the image and the orthophoto captured by the UAV.
Step (2) Calculate the transform matrix based on the perspective transformation [31].
Step (3) Transform the image into the coordinates of the orthophoto using the transformation matrix.
Step (4) Determine the length of the front of the vehicle according to the horizontal and vertical movement when the vehicle is located in the intersection.

**Figure 3:**
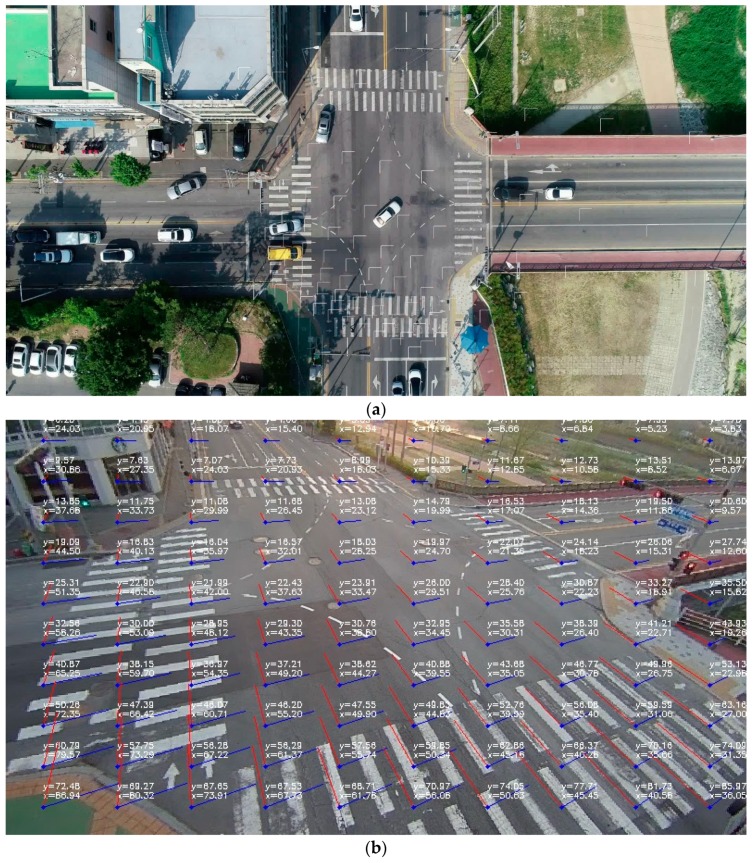
Reference dataset for the determination of vehicle trajectory: (**a**) orthophoto by unmanned aerial vehicle (UAV); (**b**) reference dataset.

Figure 3a shows the original orthophoto of the study area, and Figure 3b represents the calibrated image, including the frontal widths of the vehicles according to the horizontal and vertical movements, which were determined using the perspective transformation. Since all vehicles have similar frontal widths, it is possible to assume the width of the frontal surface when the vehicle is moving in the horizontal or vertical direction at a specific position of the acquired image. These estimates can serve as the initial value for correcting the frontal width when calculating the exact bounding box of a vehicle. In Figure 3b, red lines (y-axis values) represent the value of the width of the front side of a vehicle moving in the horizontal direction, and blue lines (x-axis values) represent the initial value of the front-side width of a vehicle moving in the vertical direction.

## 3. Proposed Methodology for The Determination of Vehicle Trajectory

**Figure 4:**
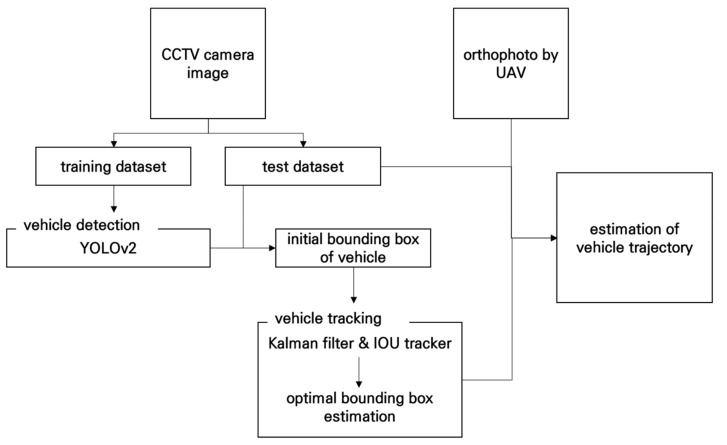
The framework of the proposed methodology.

In this manuscript, to determine the vehicle trajectory, we propose an algorithm that consists of vehicle detection and trajectory estimation and correction (Figure 4). The details are as follows.

### 3.1. Vehicle Detection Using YOLO

**Figure 5:**
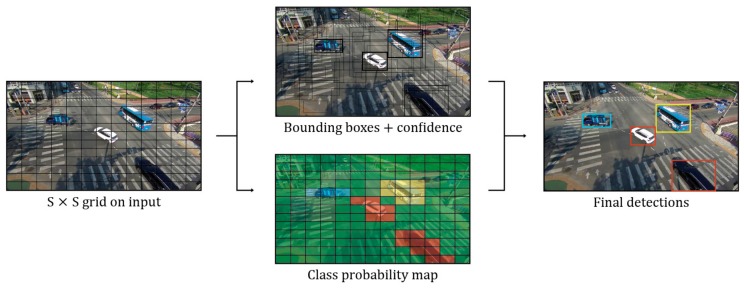
The framework of YOLO on our methodology.

As mentioned in the introduction section, the traditional algorithms based on image processing have various disadvantages: they cannot distinguish overlapping vehicles, and they are not able to detect vehicles when the weather changes or when there are shadows. Recently, due to the development of deep-learning techniques and the increase in training datasets, object-detection techniques have been improved. The proposed method is intended for detecting and tracking a moving vehicle in real time. Therefore, we tried to detect vehicles using the YOLOv2 method, which exhibits high speed and relatively high accuracy among the many deep-learning algorithms [32]. A network called YOLOv1, which is the original version of YOLO, assumed end-to-end object detection as a regression problem [33]. YOLOv1 divides the input image into S × S grids and predicts the probability of B (bounding box) and C (class) for each grid, as shown in Figure 5.

**Figure 6:**
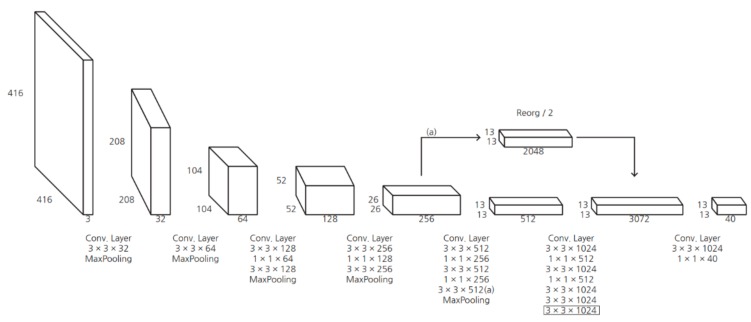
The architecture of YOLOv2.

Each bounding box consists of the predicted object size and the center position of the predicted object. These values are expressed as a percentage of the total image size. The object confidence represents the reliability of the object existing in the box, which is defined as Equation (2) [27,32]:

(2) $$\mathrm{Confidence}=\mathrm{Pr}\left(object\right)\times \;IOU_{pred}^{truth}$$

where $\mathrm{Pr}\left(object\right)$ represents the probability of the object existing in the current grid and $IOU_{pred}^{truth}$ represents the IOU between the predicted box and true box. Most bounding boxes below the threshold will be removed. In addition, redundant bounding boxes were removed by applying the non-maximum suppression (NMS) method [34]. The use of YOLOv2 in the proposed algorithm has the advantages of decreased computational costs, increased speed, and increased mean average precision (mAP) compared to YOLOv1. Batch normalization is used to preprocess the input data, which greatly improves mAP performance [35]. In addition, the anchor mechanism of Faster R-CNN and k-mean clustering is used to obtain the anchor box [32,35]. The YOLOv2 network structure is shown in Table 2 and Figure 6.

### 3.2. Trajectory Estimation by Kalman Filtering and IOU Tracker

YOLOv2 has a relatively high vehicle-detection accuracy compared to other CNN-based algorithms. Nevertheless, the position of a vehicle may not be accurately detected, which can result large errors in detecting the trajectory of a vehicle. In addition, if a frame is missing or the result of object detection is missing, the trace will fail. To overcome these drawbacks, the proposed algorithm applies the Kalman filter to correct the tracking results. The Kalman filter can be applied when there is a stochastic error in the measured value of an object and when the state at a specific point of the object has a linear relationship with the state at the previous point [36,37]. If there are errors in the continuously measured values, the position of the object can be accurately estimated using the Kalman filter. Based on Reference [36], the workflow of the Kalman filter is as follows when the initial value ${\hat{x}}_{0}$ and initial covariance $P_{0}$ are based on the measured value $z_{k}$ [36,37,38].

Step 1) State prediction: for each time step k, a prediction ${\hat{x}}_{\bar{k}}$ of the state at that time step was made using Equation (3):

(3) $${\hat{x}}_{\bar{k}}=Ax_{k-1}+Bu_{k}$$

where $x_{k-1}$ is a vector representing the process state at time k − 1, A is the state transition matrix, and B is the control input matrix [36].

Step 2) Error covariance prediction: the time-updating steps achieved by projecting the estimated error covariance are applied using Equation (4):

(4) $$P_{\bar{k}}=AP_{k-1}A^{T}+Q$$

where $P_{k-1}$ represents an error covariance matrix in the state prediction at time k − 1 and Q represents the noise covariance.

Step 3) Kalman gain: the gain of the Kalman filter is computed to calculate the estimated values by using Equation (5):

(5) $$K_{k}=P_{\bar{k}}H^{T}{\left(HP_{\bar{k}}H^{T}+R_{k}\right)}^{-1}$$

where H is the matrix for converting state space and R is measurement noise covariance [36].

Step 4) State/error covariance update: using Kalman gain, the predicted measurement and error covariance matrix are updated using Equation (6):

(6) $${\hat{x}}_{k}={\hat{x}}_{\bar{k}}+K_{k}\left(z_{k}-H{\hat{x}}_{\bar{k}}\right)\;P_{k}=\left(I-K_{k}H\right)P_{\bar{k}}$$

Steps 1–4 are applied iteratively until the predicted values converge. In this manuscript, four variables for the x, y coordinates and speed of the vehicle to the x- and y-axes were calibrated using the Kalman filter. Then, the IOU tracker was used to track the trajectory of each vehicle. If the vehicle detected in the current frame by the YOLOv2 algorithm is also present in the previous frame, it should be maintained that the two objects are the same. When cameras are used to continuously capture a vehicle over small time intervals, the probability of the position and size of each vehicle overlapping in adjacent frames is very high. Based on this, we used the IOU tracker algorithm to track the vehicle [39]. The IOU tracker algorithm compares the detected vehicle objects in the next frame with the location and size of the vehicle object in every frame and builds the idea that the vehicle with the largest IOU is the same as the vehicle in the previous frame. The feature of this algorithm is that if objects have few differences between frames, they can achieve high tracking performance using a small amount of computation.

### 3.3. Trajectory Correction using Determination of Optimal Bounding Box

**Figure 7:**
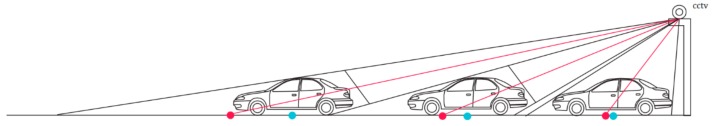
The center position of the moving vehicle resulting from the geometric displacement of the camera.

The bounding box of moving vehicles obtained by YOLOv2 causes a positional error resulting from the position and angle of the CCTV camera, despite the fact that the trajectory of a moving vehicle is corrected by Kalman filtering and IOU tracker. Therefore, we proposed a new algorithm to estimate the absolute position of a moving vehicle more accurately. The key to the proposed method is to calculate the angle corresponding to the moving direction of the vehicle using the bounding box of the vehicle extracted in each frame. Then, the position of the vehicle is corrected by calculating the amount of change in the frontal angle and length of the moving vehicle. Figure 7 shows an example of the positional error caused by the geometric displacement of the camera. To track the correct vehicle trajectory, we should extract the blue point as the center point of the moving vehicle. However, the red point could be extracted due to impacts from camera geometry. The position of the vehicle with respect to the horizontal direction has the same geometric characteristic, but the geometric characteristic changes with the distance of the vehicle in the vertical direction of the camera. Therefore, the error occurs in only the vertical direction with respect to the position of the camera.

**Figure 8:**
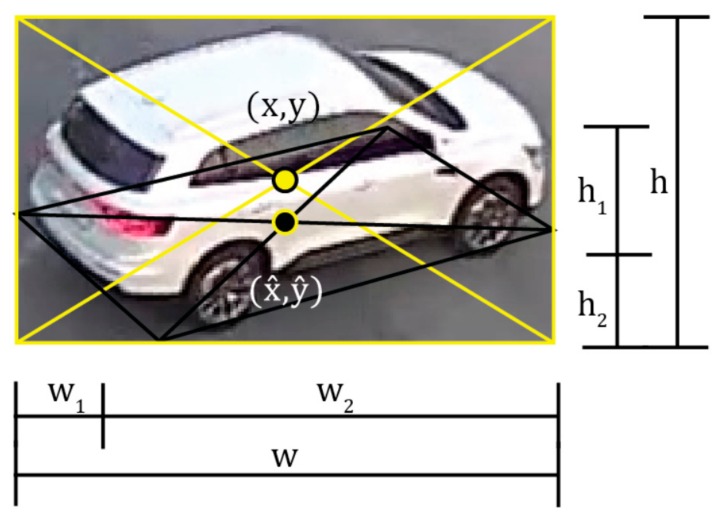
The relationship between the geometric parameters of the moving vehicle.

The relationship between the center position of the vehicle and each geometric parameter based on the bounding box is defined as shown in Figure 8. In Figure 8, the center of the bounding box (x, y) does not reflect the actual center position $\left(\hat{x},\hat{y}\right)$ due to the camera’s geometric displacement. Therefore, when the (x, y) coordinates are used as the trajectory, an error may occur in the vertical direction. Therefore, in this manuscript, the optimal bounding box of (w_1_, w_2_, h_1_, h_2_) is calculated for the initial bounding box by (w, h) by YOLOv2 and the Kalman filter.

At first, using YOLOv2, $\left(x,y\right)$ coordinates, width and height (w,h) of the bounding box for a moving vehicle are determined at every frame. Because the location of the vehicle object determined by YOLOv2 has relatively low accuracy, the coordinates of the vehicle are corrected using a Kalman filter. Define $\left(x_{0},y_{0},w_{0},h_{0}\right)$ as the initial $\left(x,y\right)$ coordinate; width and height of the bounding box of a moving vehicle are calculated by YOLOv2 and the Kalman filter. The initial vehicle information $\left(x_{i+1},y_{i+1},w_{i+1},h_{i+1}\right)$ of the bounding box for the moving vehicle in the (i + 1)th frame is updated through the following process. The side direction of the moving vehicle $\theta_{side}$ is calculated by using the coordinate difference of the bounding box between the previous frame and the current frame using Equation (7):

(7) $$\theta_{side}=\mathrm{atan}(\frac{y_{i+1}-y_{i}}{x_{i}-x_{i+1}})$$

It is possible to estimate whether the vehicle is moving in the horizontal or vertical direction through the difference between the initial coordinate position where the vehicle is located and the frame. Using the reference dataset generated from Figure 3b, we can calculate the initial side and frontal directions for the position when the vehicle moves horizontally or vertically as Equation (8):

(8) $$(\theta_{ref\_side},\theta_{ref\_front})=\left\{\begin{array}{c} (\theta_{ref\_ver},\;\theta_{ref\_hor})\;if\;\;\theta_{ref\_ver}-\theta_{side}<\theta_{ref\_hor}-\theta_{side}\\ (\theta_{ref\_hor},\;\theta_{ref\_ver})\;if\;\;\theta_{ref\_ver}-\theta_{side}>\theta_{ref\_hor}-\theta_{side} \end{array}\right.$$

In Equation (8), $\theta_{ref\_side}$ and $\theta_{ref\_front}$ represent the direction of the side and front of the vehicle according to the reference coordinates of the calibrated camera. The frontal angle $\theta_{front}$ of a moving vehicle is estimated by calculating the amount of change in the frontal angle of the vehicle using the ratio of the amount of change in the traveling direction angle:

(9) $$\theta_{front}=\theta_{ref\_front}+\theta_{\delta \_front}=\theta_{ref\_front}+\frac{\theta_{ref\_side}\times \left(180{}^{\circ}-abs\left(\theta_{ref\_side}-\theta_{ref\_front}\right)\right)}{abs\left(\theta_{ref\_side}-\theta_{ref\_front}\right)}$$

In addition, the initial side and frontal length ($l_{ref\_side}$ and $l_{ref\_front}$) of a moving vehicle can be determined as Equations (10) and (11):

(10) $$l_{ref\_side}=\left\{\begin{array}{c} l_{ref\_ver},\;\;if{\;\theta }_{ref\_side}=\theta_{ref\_ver}\;\\ l_{ref\_hor},\;if\;{\;\theta }_{ref\_side}=\theta_{ref\_hor} \end{array}\right.$$

(11) $$l_{ref\_front}=\left\{\begin{array}{c} l_{ref\_ver},\;\;if{\;\theta }_{ref\_front}=\theta_{ref\_ver}\;\\ l_{ref\_hor},\;if\;{\;\theta }_{ref\_front}=\theta_{ref\_hor} \end{array}\right.$$

Then, the front length $l_{front}$ of the vehicle in the image may change as the vehicle rotates due to geometric displacement of the calibrated camera. The front length $l_{front}$ is based on the reference length $l_{ref\_front}$ according to the initial length using the average frontal length of vehicles at the specific position of the calibrated image (Figure 4b). The $l_{front}$ can be estimated using the assumption that the variation in $l_{\delta \_front}$ is equal to the ratio of the variation in the frontal angle of the vehicle as in Equation (12):

(12) $$l_{front}=l_{ref\_front}+l_{\delta \_front}=l_{ref\_front}+\frac{\left(l_{ref\_side}-l_{ref\_front}\right)\times \theta_{\delta \_front}}{\theta_{front}}$$

**Figure 9:**
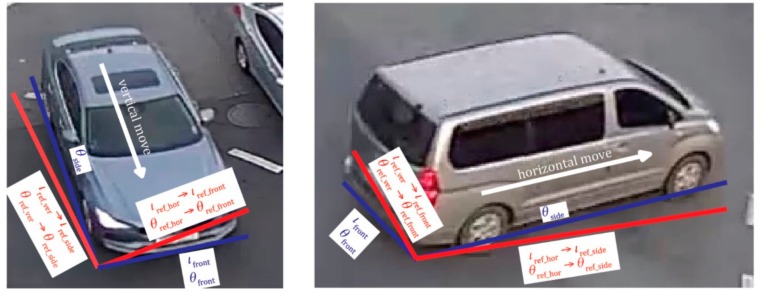
The relationships between the geometric parameters of a moving vehicle.

Finally, the corrected coordinates $\left({\hat{x}}_{i+1},{\hat{y}}_{i+1}\right)$ of the center position of the vehicle are calculated using $\theta_{front}$, $l_{front}$, and the width and height of the bounding box for the initial vehicle, as shown in Figure 9. $\left({\hat{x}}_{i+1},{\hat{y}}_{i+1}\right)$ can be calculated using Equation (13).

$${\hat{x}}_{i+1}=x_{i+1}$$

(13) $${\hat{y}}_{i+1}=y_{i+1}+\frac{h}{2}-\frac{l_{front}\times \sin\left(\theta_{front}\right)+(w-\left(l_{front}\times \cos\left(\theta_{front}\right))\right)\times \tan\left(\theta_{side}\right)}{2}$$

As shown in Figure 8, geometric distortion does not occur in the horizontal direction with respect to the moving direction of the vehicle. Since distortion occurs in the position of the vehicle in only the vertical direction, the distortion in the vertical direction can be corrected using Equation (13). Figure 9 shows an example of the optimal bounding box of the vehicle estimated using Equations (7)–(13). As shown in the blue lines of Figure 9, the moving direction and the front length of the vehicle in the bounding box can be accurately estimated using the initial information (red line) of $\theta_{ref\_ver}$, $\theta_{ref\_hor}$, $l_{ref\_ver}$, and $l_{ref\_hor}$, of the calibrated camera. Through these parameters, the exact center position of the vehicle can be calculated using the geometric characteristics of the bounding box in Figure 8.

## 4. Results and Discussion

### 4.1. Training and Test Datasets

**Figure 10:**
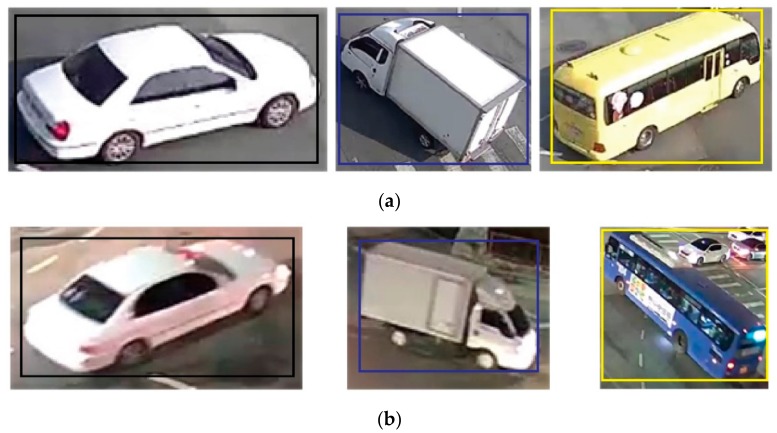
Example of the training dataset: **(a)** daytime images; (**b**) nighttime images.

It is necessary to generate training data to estimate the initial bounding box of a vehicle using the YOLOv2 model. The YOLOv2 model for vehicle detection classifies the vehicles as a car, truck, or bus. Training datasets that include vehicle images and the coordinates of a bounding box corresponding to each vehicle were generated using daytime and nighttime images obtained using a total of four cameras. A total of 145,414 (car: 138,077, truck: 6429, and bus: 908) training data was generated. An example of the training data is shown in Figure 10. In Figure 10, the bounding boxes indicate the locations of each vehicle.

The reference dataset for evaluating the performance of the proposed technique was generated by extracting the location information for the vehicles by using the frame of the video data from the intersection that was collected by a UAV, as shown in Figure 3a. The quantitative estimation was applied using the root mean squared error (RMSE) between the results obtained by the proposed algorithm and the true coordinates obtained by the UAV. Meanwhile, the algorithm developed in this manuscript needs real-time processing for practical application to ITS using CCTV cameras. Therefore, the acquired image was converted to a low resolution and applied in the experiment. It is also possible to evaluate whether the algorithm can be applied to images captured by a low-resolution camera.

### 4.2. Experimental Results and Analysis

**Figure 11:**
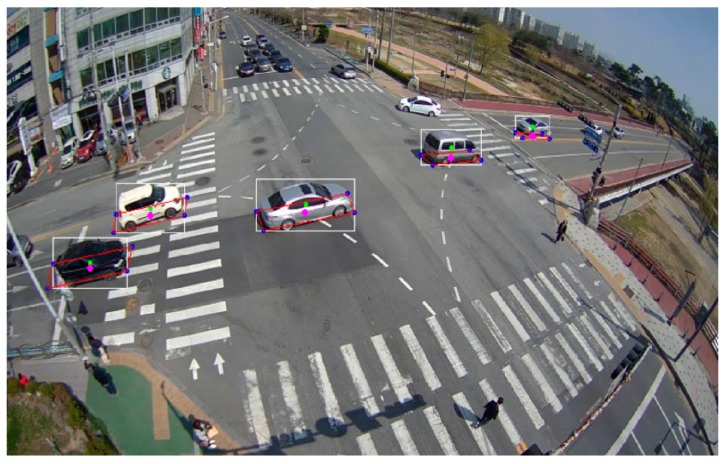
Example of the extracted bounding boxes of moving vehicles.

The center coordinates of the vehicle obtained through the proposed method are compared with the center point of the bounding box extracted using the conventional algorithm. In this manuscript, the conventional algorithm refers to the optimized framework using a Kalman filter and IOU tracker for object tracking based on YOLOv2 for object detection. In the proposed algorithm, trajectory correction using the determination of the optimal bounding box was added. Figure 11 shows an example of the results of the proposed algorithm (pink point) and YOLOv2 (green point). The bounding box extracted by the proposed algorithm is represented by the bounding box in red. As shown in Figure 11, the center points extracted by the proposed algorithm reflect the center of momentum more effectively than those extracted by the conventional algorithm. In addition, the center of the actual lane-travel path is well represented.

**Figure 12:**
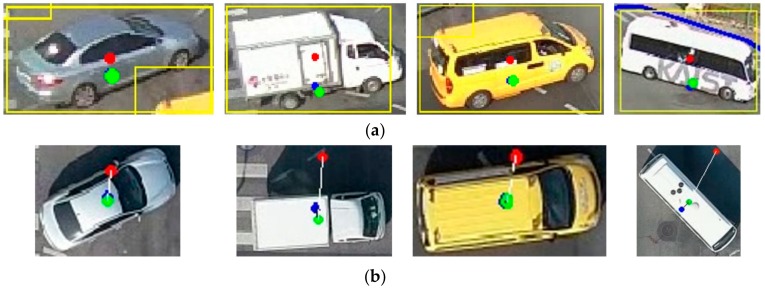
The projected center points of moving vehicles in closed-circuit television (CCTV) camera images and orthophotos: (**a**) CCTV camera images; (**b**) orthophoto images. (Green point: ground truth, blue point: results of the proposed algorithm, and red point: results of the conventional algorithm).

To analyze the results in detail, the location information of the extracted vehicle was projected onto the orthoimage taken at the same time and analyzed. Figure 12a describes the center point of each vehicle as extracted by the proposed method (blue point) and traditional algorithm (red point) relative to the ground truth (green point) in the CCTV camera image, while Figure 12b presents the center points projected onto the orthoimages corresponding to the results in Figure 12a. As shown in Figure 12, the center coordinates of the vehicles extracted by the proposed method are closer to the ground truth. In particular, the center points of the bounding boxes obtained using the existing YOLOv2 method are outside of the vehicle because traditional algorithms do not consider the geometric characteristics of the center points of vehicles in the CCTV camera images. As shown in Figure 12a, the results of the conventional algorithm include errors in the direction of specific axes, but the results of the proposed technique adjusted using Equation (13) indicate that these errors were corrected.

**Figure 13:**
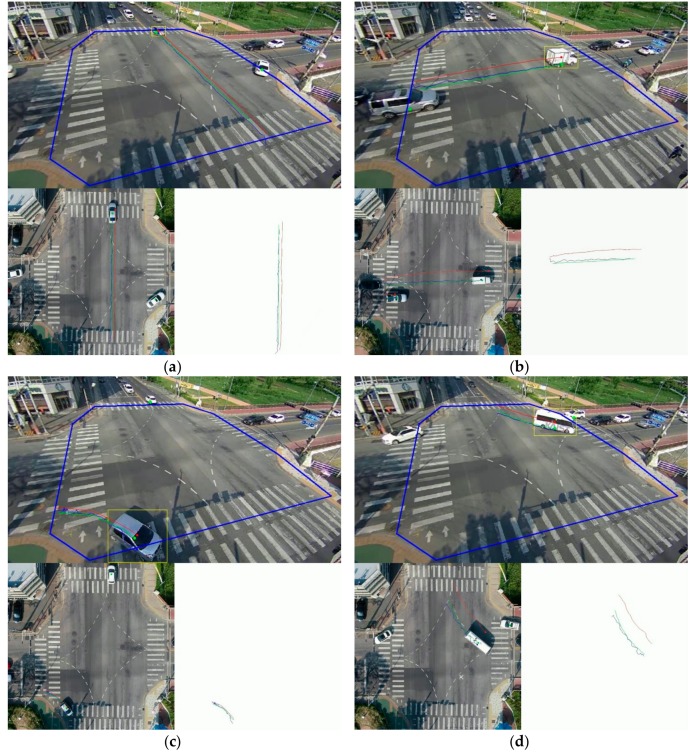
Results of the vehicle trajectory extraction corresponding to the different algorithms: (**a**) driving straight (up and down); (**b**) driving straight (left and right); (**c**) right turn; and (**d**) left turn.

Figure 13 shows an example of the vehicle-trajectory extraction results estimated using the bounding boxes of vehicles extracted according to the frame and center coordinates of the vehicles corresponding to four cases. The yellow box indicates the initial bounding box extracted through object detection. The green line is the reference trajectory, and the red line indicates the estimated result achieved using the conventional algorithm. The blue line is the trajectory extracted through the proposed method. As shown in Figure 13, the trajectory extracted by the proposed method shows a path similar to the moving lane of the actual vehicle. When the vehicle moves in a straight line (Figure 13a,b), the trajectory of the vehicle indicated by the conventional algorithm includes a bias compared with the ground truth, but the result provided by the proposed technique tends to be very similar to the ground truth. Similar trends were seen when the vehicle turned left or right (Figure 13c,d). However, it was confirmed that an error occurred relative to the result for the straight line. This is because the tracking of the object in the frame units could not accurately reflect the trend of the curve. Nevertheless, it can be seen that the results of the proposed technique are quite close to the ground truth.

To perform a quantitative evaluation, the center positions of 6312 vehicles in each frame were extracted and the RMSE was calculated by determining the differences from the ground-truth positions. As shown in Table 3, the RMSE of the y-axis is larger than the RMSE of the x-axis. This result means that the error within the lane where the vehicle is traveling is larger than that in the direction of the relative path. Nevertheless, the proposed scheme has a lower RMSE than the conventional algorithm. Therefore, it is possible to extract the location information for a vehicle at the intersection more effectively using the technique developed in this study.

## 5. Conclusions

In this manuscript, a new method for automatically estimating vehicle trajectories is proposed using the YOLOv2, a Kalman filter, an IOU tracker, and the trajectory correction method for moving vehicles according to the geometric displacement from a calibrated camera. The trajectory and accurate information on the center position of a vehicle are extracted using images acquired from a camera installed at an intersection based on image processing techniques. First, real-time vehicle object detection is performed using the YOLOv2 model to determine the initial vehicle location and bounding box, which are used to define the geometric characteristics of the vehicle. Then, to correct the vehicle location extracted by YOLOv2, the trajectory was calibrated using the Kalman filter and IOU-tracker algorithm. Finally, the accurate position of a moving vehicle is determined using the basic angle and the length of the front of the vehicle according to the position of the vehicle. In particular, positional error resulting from the geometric displacement of the calibrated camera is revised in the proposed algorithm. In the quantitative and visual estimation, the proposed method can detect the center points of moving vehicles better than the conventional algorithm. Although the center points of the bounding boxes obtained using the existing algorithm are often outside the vehicle due to various effects, such as relief displacement of the vehicle, the proposed algorithm can minimize this error.

## Figures and Tables

**Table 1:** Description of XNO-6010R.

Sensor	XNO-6010R
Imaging devices	1/2.8’’ 2-megapixel CMOS
Effective pixels	1945 (H) × 1097 (V), 2.13 megapixel
Signal-to-noise ratio	50 dB
Focal length	2.4 mm fixed
Field of view	Horizontal: 139.0° Vertical: 73.0° Diagonal: 167.0°
Weight	1.22 kg
Installation height	9.5 m

**Table 2:** The network structures of YOLOv2.

Num	Type	Input	Filters	Size/Stride	Output
0	conv	416 × 416 × 3	32	3 × 3/1	416 × 416 × 32
1	max	416 × 416 × 32		2 × 2/2	208 × 208 × 32
2	conv	208 × 208 × 32	64	3 × 3/1	208 × 208 × 64
3	max	208 × 208 × 64		2 × 2/2	104 × 104 × 64
4	conv	104 × 104 × 64	128	3 × 3/1	104 × 104 × 128
5	conv	104 × 104 × 128	64	1 × 1/1	104 × 104 × 64
6	conv	104 × 104 × 64	128	3 × 3/1	104 × 104 × 128
7	max	104 × 104 × 128		2 × 2/2	52 × 52 × 128
8	conv	52 × 52 × 128	256	3 × 3/1	52 × 52 × 256
9	conv	52 × 52 × 256	128	1 × 1/1	52 × 52 × 128
10	conv	52 × 52 × 128	256	3 × 3/1	52 × 52 × 256
11	max	52 × 52 × 256		2 × 2/2	26 × 26 × 256
12	conv	26 × 26 × 256	512	3 × 3/1	26 × 26 × 512
13	conv	26 × 26 × 512	256	1 × 1/1	26 × 26 × 256
14	conv	26 × 26 × 256	512	3 × 3/1	26 × 26 × 512
15	conv	26 × 26 × 512	256	1 × 1/1	26 × 26 × 256
16	conv	26 × 26 × 256	512	3 × 3/1	26 × 26 × 512
17	max	26 × 26 × 512		2 × 2/2	13 × 13 × 512
18	conv	13 × 13 × 512	1024	3 × 3/1	13 × 13 × 1024
19	conv	13 × 13 × 1024	512	1 × 1/1	13 × 13 × 512
20	conv	13 × 13 × 512	1024	3 × 3/1	13 × 13 × 1024
21	conv	13 × 13 × 1024	512	1 × 1/1	13 × 13 × 512
22	conv	13 × 13 × 512	1024	3 × 3/1	13 × 13 × 1024
23	conv	13 × 13 × 1024	1024	3 × 3/1	13 × 13 × 1024
24	conv	13 × 13 × 1024	1024	3 × 3/1	13 × 13 × 1024
25	route	16th			26 × 26 × 512
26	reorg	26 × 26 × 512		_×_/1	13 × 13 × 2048
27	route	26th and 24th			13 × 13 × 3072
28	conv	13 × 13 × 3072	1024	3 × 3/1	13 × 13 × 1024
29	conv	13 × 13 × 1024	40	1 × 1/1	13 × 13 × 40

**Table 3:** Root mean squared error (RMSE) of vehicle trajectories corresponding to the different vehicle detection algorithms.

	Conventional Algorithm	Proposed Algorithm
RMSE (X)	14.35	7.41
RMSE (Y)	30.00	14.75
RMSE	33.25	16.51
